# Genomic Inverse PCR for Exploration of Ligated Breakpoints (GIPFEL), a New Method to Detect Translocations in Leukemia

**DOI:** 10.1371/journal.pone.0104419

**Published:** 2014-08-19

**Authors:** Elisa Fueller, Daniel Schaefer, Ute Fischer, Pina F. I. Krell, Martin Stanulla, Arndt Borkhardt, Robert K. Slany

**Affiliations:** 1 Department of Genetics, Friedrich Alexander University, Erlangen, Germany; 2 Department of Pediatric Oncology, Hematology and Clinical Immunology, University Children's Hospital, Medical Faculty, Heinrich Heine University, Düsseldorf, Germany; 3 Department of Pediatric Hematology and Oncology, Hannover Medical School, Hannover, Germany; Georg Speyer Haus, Germany

## Abstract

Here we present a novel method “Genomic inverse PCR for exploration of ligated breakpoints” (GIPFEL) that allows the sensitive detection of recurrent chromosomal translocations. This technique utilizes limited amounts of DNA as starting material and relies on PCR based quantification of unique DNA sequences that are created by circular ligation of restricted genomic DNA from translocation bearing cells. Because the complete potential breakpoint region is interrogated, a prior knowledge of the individual, specific interchromosomal fusion site is not required. We validated GIPFEL for the five most common gene fusions associated with childhood leukemia (MLL-AF4, MLL-AF9, MLL-ENL, ETV6-RUNX1, and TCF3-PBX1). A workflow of restriction digest, purification, ligation, removal of linear fragments and precipitation enriching for circular DNA was developed. GIPFEL allowed detection of translocation specific signature sequences down to a 10^−4^ dilution which is close to the theoretical limit. In a blinded proof-of-principle study utilizing DNA from cell lines and 144 children with B-precursor-ALL associated translocations this method was 100% specific with no false positive results. Sensitivity was 83%, 65%, and 24% for t(4;11), t(9;11) and t(11;19) respectively. Translocation t(12;21) was correctly detected in 64% and t(1;19) in 39% of the cases. In contrast to other methods, the characteristics of GIPFEL make it particularly attractive for prospective studies.

## Introduction

The realization that certain subtypes of leukemia are invariably associated with recurrent genomic abnormalities was a seminal discovery in leukemia research. This was first recognized in conjunction with chronic myeloid leukemia and the paradigmatic Philadelphia chromosome [1]. Nowadays we know that this is a widespread phenomenon. The determination of genotype has become essential for diagnosis, stratification, treatment planning and prognosis of hematological malignancies. Particularly in infant and childhood leukemia almost half of all diagnosed cases are characterized by the persistent appearance of distinctive chromosomal translocations [2].

Because of the importance of these genetic markers for clinical management a series of methods has been devised that allows the detection of the underlying genetic lesion. Cytogenetics and fluorescent in situ hybridization (FISH) are generally applied to demonstrate the presence and overall structure of genomic alterations. However, both approaches require mitotic cells, cumbersome experimental procedures and experienced operators for success. Alternative methods using archived genetic material have also been developed. Since most translocations create in-frame fusion proteins there are only a limited number of exons within both fusion partners that can be joined productively. This fact has been exploited by PCR based methods that use RNA/cDNA as template [3], [4]. In this way the number of primer pairs necessary to interrogate for the presence of a specific translocation is limited and the expected amplification products can be predicted. The drawback is the labile nature of RNA that often precludes successful amplification from stored or aging samples. To avoid this problem DNA based methods have been explored [5], [6]. Yet, the actual genomic breakpoints are usually unknown and they are distributed over a large stretch of intronic sequences. This mandates either the use of an unwieldy number of different primer pairs or long range PCR strategies with the disadvantage of non-quantifiable amplicons of unknown length that may well exceed the practicable limits of current PCR.

**Figure 1:**
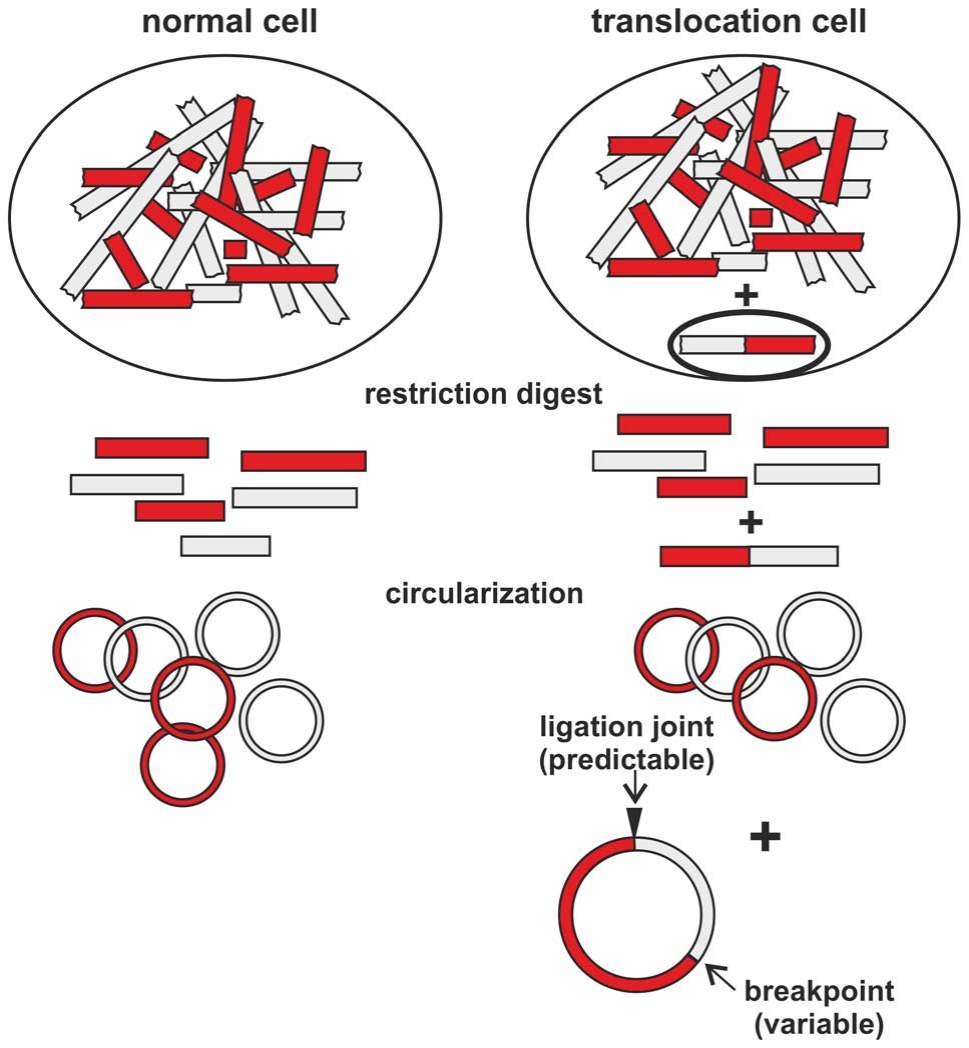
Basic principle of GIPFEL. Upon restriction digest and circularization of genomic DNA only genomic DNA from translocation bearing cells will form circles that join DNA of two different chromosomes. The junction is predetermined by the location of the genomic breakpoint. By probing for all possible ligation junctions with PCR the presence of a translocation can be ascertained.

To avoid these pitfalls, we devised a novel method that can detect chromosomal translocations at the DNA level creating constant, predictable, and quantifiable amplicons. This technique, that we called GIPFEL (genomic inverse PCR for exploration of ligated breakpoints) utilizes the fact that genomic breakpoints are usually confined to defined chromosomal regions. Restriction digest of genomic DNA followed by circularization of resulting fragments will divide even large breakpoint regions into a manageable number of DNA circles. Only cells with translocations will create a “signature” circle that is uniquely characteristic for the nature of the underlying genomic aberration (figure 1). These circles can be quantified by real-time PCR because the sequence of the corresponding ligation joint can be derived from the known genomic sequence and the respective location of the restriction sites within the breakpoint region. Hence corresponding amplicons of suitable size for real-time PCR can be designed. Positive amplification results do not only reveal the presence of a translocation but they also give topical information of the approximate localization of the genomic break. By selecting appropriate restriction enzymes even large breakpoint regions can be covered with relatively few primer/PCR reactions. Here we demonstrate proof-of-principle experiments testing GIPFEL on the five most frequent translocations in childhood leukemia t(4;11), t(9;11), t(11;19), t(12;21), and t(1;19).

## Materials and Methods

### Circularization of genomic DNA

Genomic DNA from clinical repositories was provided pre-purified. Samples were collected with written informed consent and all institutional and national guidelines for employing human material in research were observed. Patients were enrolled in multicenter trial AIEOP-BFM ALL 2000 on treatment of childhood ALL. Diagnosis, characterization and treatment of ALL were performed as previously described [7], [8]. The trial was approved by the institutional review board of Hannover Medical School, Hannover, Germany. Written informed consent for the use of specimen for research was obtained from all study individuals, parents or legal guardians and approved by the institutional review board.

All enzymes used in the procedure were obtained from New England Biolabs (Frankfurt/Main, Germany) and used with the appropriate buffers recommended by the manufacturer. For cell lines and buffy coats DNA was prepared from 1 to 5×10^6^ cells with the QIAampDNA Blood Mini Kit exactly according to the instructions of the manufacturer (Qiagen, Hilden, Germany).

If available, e.g. from cell lines, GIPFEL started with 2.5 µg of DNA corresponding to approximately 3.8×10^5^ genome equivalents (calculating with 6.6 pg DNA per cell). For detection of translocations in repository DNA, the nucleic acids were either pre-amplified with REPLI-g Ultra Fast Mini Kit according to the manufacturer's (Qiagen) instructions or, when probing for MLL translocations, only 1 µg stored DNA was used directly. The DNA was incubated either with 200 units BamHI-HF (for MLL translocations) or with 200 units of SacI-HF or MfeI-HF for detection of t(12;21) and t(1;19), respectively. Reactions were set up in 100 µl volume using the buffer recommended by the manufacturer and digests were performed for 2 h.

Restriction fragments were isolated by addition of 500 µl buffer PB (Qiagen) to the digestion reaction and a subsequent purification on QIAquick gel extraction columns (Qiagen) according to the instructions of the manufacturer. To improve recovery of longer fragments elution was done with 50 µl of deionized water pre-warmed to 60°C and columns were incubated for 5 minutes at 60°C before final centrifugation.

Religation was performed for 2 h at 24°C in a 100 µl reaction using the total column eluate and 2 µl (800 units) of T4-DNA ligase and the appropriate buffer. After ligation linear DNA fragments were digested by addition of 1 µl (100 units) of exonuclease III and incubation for 30 min at 37°C with a subsequent 5 min heat inactivation at 95°C.

Enriched circular DNA was concentrated by standard alcohol precipitation.

### Primer design and semi-nested real time PCR

*In silico* predictions were done deriving the sequences of all possible ligation junctions that would be created from religation of a genomic fragment carrying a chromosomal breakpoint. Primers spanning ligation sites were designed to generate amplicons suitable for real time PCR (see table 1 and table S1) (https://eu.idtdna.com/analyzer/Applications/OligoAnalyzer/) [9]. To restrict the number of PCRs necessary to include the complete breakpoint region sometimes closely spaced (<1 kb) restriction sites were covered only by a single primer.

**Table 1:** Primers used for GIPFEL.

Name	detection	sequence(5′-3′)	GC %	T_M_ °C	l.	size PCR product
**MLL**						
**MLL-B1r.4**	MLL outer pr.	GCTTTCGTGGAGGAGGCTCAC	61.9	69.5	21	
**MLL-B1r-n**	MLL inner pr.	CTGCTTTTCTTTGGGGCAGGATC	52.2	62.4	23	
**MLL-B2f.4**	MLL control pr.	TGGGTGAGTTATACACATGATGC	43.4	63.5	23	301
**AF4**						
**AF4-B1f**	breakpoints	CTGAAGATGCCTTCTCAGTCAG	50	60.3	22	361
**AF4-B2f**		TGTGGATTCTTTACTCCCTGTCC	47.8	60.6	23	336
**AF4-B3f**		GCCACACCATGTGCAGAGACC	61.9	63.7	21	402
**AF4-B4f.2**		CTTATAGTAGCCCAAGAGGAAAG	43.5	58.9	23	219
**AF4-B6f**		GTGTGTGTGCTTGTAGTCTTAGC	47.8	60.6	23	426
**AF4-B7f**		TTGTTCTATTGATTCACCTTCGAC	37.5	63.0	24	257
**AF4-B8f**		GTATGGCAGGCATTGCATCCAC	54.5	70.5	23	265
**AF9**						
**AF9-B1f.2**	breakpoints	TGTTTGTATTTTGCTTGTGTAAAGG	32	62.7	25	199
**AF9-B2f.3**		GTAATTTAATATAGATTATTGCAGG	24	54.1	25	169
**AF9-B3f**		ACAGTACAACCATCCAAGTCAGG	47.8	60.6	23	462
**AF9-B4f**		AGTGGACAAGATAAGAAGGCTCC	47.8	60.6	23	281
**AF9-B5f**		GTACCTGGCACATAGTTGGTAG	50	60.3	22	429
**AF9-B6f.2**		CCCACTGGAATGTCACGTTAGG	54.5	67.5	22	183
**AF9-B7f**		TGTCTTTAAGGAATGGAAAACTGC	37.5	57.6	24	470
**AF9-B8f**		GAGGAATTACAGCTCTGAGCCC	54.5	62.1	22	287
**AF9-B9f**		TCGCTAGTGCATAGATTGTTAGG	43.5	58.9	23	318
**AF9-B10f**		GTTGTACCAGTTACAGTTCAACTG	41.6	59.2	24	317
**ENL**						
**ENL-B6f**	breakpoints	GAGCTCCTCTGACTCCCTAGG	61.9	63.4	21	337
**ENL-B7f**		CTCTGCCTTCTTCTTGGGAACC	54.5	67.1	22	369
**ENL-B8f.2**		CTCTCTGGACTCCTCTTAATACC	47.8	59	23	243
**ENL-B9f**		CACTTAGTGCTATGAAGGCGTTG	47.8	60.6	23	324
**ENL-B11f**		ACTTTGCCGTGGAAGTCAATCC	50	60.3	22	286
**ENL-B12f**		TGCTGTTTGCTGCTTGTCATCC	50	60.3	22	398
**ENL-B13f.2**		TCATTGCAGACTCCACCTCTCC	54.5	62.1	22	371
**ENL-B14f**		CCTAACCACAATATCATTCTGGC	43.4	63.2	23	350
**ENL-B15f.7**		CTGGGTCTGCAGTGATTGTGG	57.1	61.8	21	94
**ENL-B16f.2**		GGTGGCATCCCTCCTCGTGG	70	65.5	20	186
**ENL-B17f**		GTGGAATTCAGGGACAGTTCAG	50	60.3	22	313
**ETV6**						
**ETV6-S1r**	ETV6 outer pr.	GATGTGGTTCATGTAAGCCAGGTCTTC	48	68.2	27	
**ETV6-S1r-n**	ETV6 inner pr.	GGAGGACGCTGGGCAGTGATTATTC	56	69.1	25	
**ETV6-S2r**	ETV6 outer pr.	AAAGGGACAGTACCTCAAGGCAGAAG	50	67.9	26	
**ETV6-S2r-n**	ETV6 inner pr.	TGGCAGCACCTTGATGGTCAGCTAG	56	69.1	25	
**ETV6-S3r**	ETV6 outer pr.	GGGACATTATGCACCTGCTTGGGAG	56	69.1	25	
**ETV6-S3r-n**	ETV6 inner pr.	TAGGACTGTTCGGGGCCATCTGTC	58	68.5	24	
**RUNX1**						ETV6-S1/2/3r-n
**RUNX1-S1f**	breakpoints	CAGAGGCAAGACGGGCTGATAACC	58	68.5	24	512/444/449
**RUNX1-S2f**		AGGGACTCATGGTGACGGGAGC	64	67.9	22	196/128/133
**RUNX1-S3f**		GACTCTATATTGGAACCTCGGAAACGC	48	68.2	27	257/189/194
**RUNX1-S4f**		TTATCTGGTGGGCTGTTAGGAGGCTC	54	69.5	26	267/199/204
**RUNX1-S5f**		GGTGTGTTTCATAGGGAACTGGTTTTGC	46	68.5	28	169/101/106
**RUNX1-S6f**		CCCACACCCTAGTTTGCATCGGTTTG	54	69.5	26	131/63/68
**RUNX1-S7f**		GAGGTGGAAGTAGTCATTATGGGATAACC	45	69.1	29	670/602/607
**RUNX1-S8f**		TGGTGACAAGTTGCTTCAGGCTGATG	50	67.9	26	193/125/130
**RUNX1-S10f**		CCGGGATGACAACAGTTCAAGGAATAC	48	68.2	27	142/74/79
**RUNX1-S11f**		ACCAGGCACTTGACTCTTAGGATGTTTG	46	68.5	28	229/161/166
**RUNX1-S12f**		GTGTCATCTCAACCATGGAAAGGGTAC	48	68.2	27	323/255/260
**RUNX1-S13f**		GGAGGACCTAGTGGGATGCAAGTG	58	68.5	24	159/91/96
**RUNX1-S14f**		CTGACTGGGCAGCTCCACTATGTC	58	68.5	24	217/149/154
**RUNX1-S15f**		CCTAGTGAGTTCAGTGTGGTTTTGTCAG	46	68.5	28	174/106/111
**RUNX1-S16f**		AGTGAGCTGGGGAATCCATTCAAGTG	50	67.9	26	173/105/110
**RUNX1-S17f**		CGTTTCTAGAAGGAGTGCCGGCAG	58	68.5	24	296/228/233
**RUNX1-S18f**		GCTACCAGTCAAGTTTCCTTTCGGGC	54	69.5	26	202/134/139
**RUNX1-S19f**		AGACACAAAAGGTCAGACGCATGACAC	48	68.2	27	314/246/251
**RUNX1-S20f**		TTGGGGAGAGAAGGATGATGGTCTTG	50	67.9	26	274/206/211
**RUNX1-S21f**		AGTGGAAAAGGAGGTGGCAAGTACAG	50	67.9	26	152/84/89
**RUNX1-S22f**		AAGGAAAGAAGCTAGTTGGGGTAGCG	50	67.9	26	272/204/209
**RUNX1-S23f**		AACAGAGAAGTCGCAATAGTGCAGCAG	48	68.2	27	231/163/168
**RUNX1-S24f**		TCTCATGTTTTCCAGTTGCTTAGGCGTG	46	68.5	28	230/162/167
**RUNX1-S25f**		TGTCTTGGGGATCATTCTCGCCTGC	56	69.1	25	185/117/122
**RUNX1-S26f**		CATCAGGCAGAAAGGAAGAAGGGAAG	50	67.9	26	177/109/114
**RUNX1-S27f**		TGCAGTCACTTAGAAGCACCCATCTG	50	67.9	26	715/647/652
**RUNX1-S28f**		CAGAAAATCTTGCAGCAGTCAGCTTGC	48	68.2	27	163/95/100
**RUNX1-S29f**		TCGGTTAGCTTTCACGGAGGCAGTG	56	69.1	25	135/67/72
**RUNX1-S0f**	RUNX1 control pr.	CTTGGTTCAGAGTGTATCTCACCCTTG	48	68.2	27	404
**RUNX1-S1r**	RUNX1 control pr.	GTGAAGCCAGGGACACACACTAAATG	50	67.9	26	404
**TCF3**						
**TCF3-M1r**	TCF3 outer pr.	CTGTGCTGGAGCGGGAAGTATGC	61	68.3	23	
**TCF3-M1r-n**	TCF3 inner pr.	AGCGAGATGAGACCGCAGGAGTG	61	68.3	23	
**PBX1**						
**PBX1-M1f**	breakpoints	ACTTAAAACTTGGCCCTAGAGTCCCTC	48	68.2	27	164
**PBX1-M2f**		GTGAAGCTGAGAAAACTACATGTGTGTCG	45	69.1	29	320
**PBX1-M3f**		ATGGTGTAAGGATGGGGTGAGTGCTG	54	69.5	26	295
**PBX1-M4f**		CAAGGATGTAACCTGATGGGGAATAGTG	46	68.5	28	542
**PBX1-M5f**		TTGGTCTGTGCCTACATGTATGTGCTC	48	68.2	27	217
**PBX1-M6f**		CCAGGTGTGAGAGGCAGTGTAACATC	54	69.5	26	192
**PBX1-M7f**		CCATCTGTAAAATTGGGTGGCAGTGTAG	46	68.5	28	228
**PBX1-M8f**		TCAAGGTAAAGCTCTGAAATCCCACGC	48	68.2	27	239
**PBX1-M9f**		GATGGTGTCCCAGGAGCAAGCAAC	58	68.5	24	273
**PBX1-M10f**		GGATTGACACAGACCAAGGGGTCTTG	54	69.5	26	356
**PBX1-M11f**		AGAGAGGTCAGGAAGGGAAAGGGATG	54	69.5	26	186
**PBX1-M12f**		CGATCCCACCATTGGTCAACACAGAC	54	69.5	26	247
**PBX1-M13f**		TAGAATGAGGCAGAGCTTCCAGGATAG	48	68.2	27	224
**PBX1-M14f**		GAGAGAGACTCAGCTTCAGTAACCTG	50	67.9	26	177
**PBX1-M15f**		CCCTAGGCTGAACGAAACGAAAACTC	50	67.9	26	727
**PBX1-M16f**		TCAAAGGCAGGAGTGAGATGTCATCC	50	67.9	26	218
**PBX1-M17f**		TCTCTGACCTTCTGTCTCTGGGCAC	56	69.1	25	257
**PBX1-M18f**		CTCTGAGACACGGAACACTAGTTGTG	50	67.9	26	192
**PBX1-M19f**		TCCCTCTAGTCATATGTCTGTGCTGC	50	67.9	26	183
**PBX1-M20f**		CAAAGTATGTTGAAGTGTGTTGGCGCC	48	68.2	27	158
**PBX1-M21f**		GTACATAGGCGTTATCACCTCATTGGAAG	45	69.1	29	279
**PBX1-M22f**		GACCCCTTCTCTCTTAACTCATAATGGC	46	68.5	28	276
**PBX1-M23f**		CAGGAACAAGAACAAGAAGGCATGTAGG	46	68.5	28	199
**PBX1-M24f**		AGCATCATAGGTGACAAGGGGCCATG	54	69.5	26	164
**PBX1-M25f**		TGCCTGGTGCATGTTAAGCCTCACAG	54	69.5	26	234
**PBX1-M26f**		TAGAACATGCAGAATGCCCACCGTGG	54	69.5	26	183
**PBX1-M27f**		TGAGTGTGTTGGTACCGATGTGTGGC	54	69.5	26	147
**PBX1-M28f**		GTGAATGCCTGTGTGTACACTTAACGTG	46	68.5	28	253
**PBX1-M29f**		CTGGCGTCATAACAGAAGTAGTCACAG	48	68.2	27	268
**PBX1-M30f**		TGGCATCTGAAGCACCTGTCCTAATG	50	67.9	26	205
**PBX1-M31f**		CTGAGCTTGACCTTCCAGTCGTCTTC	54	69.5	26	204
**PBX1-M32f**		TTGGCATTGTGACCAGGAGATCTATTGC	46	68.5	28	243
**PBX1-M33f**		GATGCAAGGGAACAATTACTGGACTGTTC	45	69.1	29	346
**PBX1-M34f**		ACATTCTGAGGAAGATACATGGTTGTTCC	41	67.4	29	177
**PBX1-M35f**		TGGTGGTAATGGGGTTGGTGGGATAG	54	69.5	26	328
**PBX1-M36f**		ATACACACATGCACGTAACACCCCAAAG	46	68.5	28	167
**PBX1-M0f**	PBX1 control pr.	GCCCTGTAACCTGGGAGGTCTATTAG	54	69.5	26	298
**PBX1-M1r**	PBX1 control pr.	AACCATCTGTGGAGTGCCCGGATTAG	54	69.5	26	298

All PCR reactions were performed with BrilliantII SYBR green PCR Master Mix from Agilent Technologies (St. Clara, CA, USA) in standard 25 µl reactions using a final primer concentration of 100 nM. For first round PCR 5 µl of circularized DNA corresponding to approximately 1.9×10^5^ genome equivalents served as template. Cycle conditions were 10 min initial denaturation, followed by 22 cycles of 15 s 95°C, 30 s 64°C, 30 s 72°C for MLL translocations. Translocation t(12;21) and t(1;19) samples were pre-amplified with 25 cycles.

One µl of primary PCR product was used as input for each secondary PCR. Reactions were monitored on an optical cycler for 40 to 45 cycles under conditions as in first round PCR reaction. The multiplexing scheme is given in table 2.

**Table 2:** Multiplexing strategy for GIPFEL analysis.

**MLLAF4** *	**anchor pr.**	**#1**				**#2**			**#3**					**#4 control**
PCR1	MLL-B1r.4+	B1f+B2f+B3f				B4f.2+B6f+B7f			B8f					MLL-B2f.4
PCR2	**anchor pr.**	**#1**	**#2**		**#3**	**#4**	**#5**	**#6**	**#7**					**#8 control**
	MLL-B1r.n +	B1f	B2f		B3f	B4f.2	B6f	B7f	B8f					MLL-B2f.4
**MLLAF9** *	**anchor pr.**	**#1**				**#2**			**#3**			**#4**		**#5 control**
PCR1	MLL-B1r.4 +	B1f.2+B2f.3+B3f				B4f+B5f+B6f.2			B7f+B8f+B9f			B10f		MLL-B2f.4
	**anchor pr.**	**#1**	**#2**		**#3**	**#4**	**#5**	**#6**	**#7**	**#8**	**#9**	**#10**		**#11 control**
PCR2	MLL-B1r.n +	B1f.2	B2f.3		B3f	B4f	B5f	B6f.2	B7f	B8f	B9f	B10f		MLL-B2f.4
**MLLENL** *	**anchor pr.**	**#1**				**#2**			**#3**			**#4**		**#5 control**
PCR1	MLL-B1r.4 +	B6f+B7f+B8f.2				B9f+B11+B12f			B13f.2+B14f+15f.7			B16f.2+B17f		MLL-B2f.4
	**anchor pr.**	**#1**	**#2**	**#3**		**#4**	**#5**	**#6**	**#7**	**#8**	**#9**	**#10**	**#11**	**#12 control**
PCR2	MLL-B1r.n +	B6f	B7f	B8f		B9f	B11f	B12f	B13f.2	B14f	B15f.7	B16f.2	B17f	MLL-B2f.4
**ETV6-RUNX1**	**anchor pr.**	**#1**				**#2**			**#3**			**#4**		**#5 control**
PCR1	ETV6- S1r +S2r + S3r +	S12f + S15f + S17f + S22f + S23f + S26f + S28f				S1f + S4f + S10f + S11f + S14f + S24f + S27f			S2f + S6f + S7f + S8f + S18f + S20f + S29f			S3f + S5f + S13f + S16f + S19f + S21f + S25f		RUNX1-S0f + S1r (no anchor)
	**anchor pr.**	**#1**				**#2**			**#3**			**#4**		**#5 control**
PCR2†	ETV6- S1r-n + S2r-n + S3r-n +	S12f + S15f + S17f + S22f + S23f + S26f + S28f				S1f + S4f + S10f + S11f + S14f + S24f + S27f			S2f + S6f + S7f + S8f + S18f + S20f + S29f			S3f + S5f + S13f + S16f + S19f + S21f + S25f		RUNX1-S0f + S1r (no anchor)
**TCF3-PBX1**	**anchor pr.**	**#1**				**#2**			**#3**			**#4**		**#6 control**
PCR1	TCF3-M1r+	M1f + M6f + M12f + M13f + M26f + M29f + M33f + M36f				M4f + M8f + M9f + M10f + M16f + M21f + M23f			M2f + M11f + M20f + M25f + M28f + M34f + M35f			M3f + M5f + M14f + M18f + M19f + M24f + M32f		PBX1-M0f + M1r (no anchor)
		#5												
		M7f + M15f + M17f + M22f + M27f + M30f + M31f												
	**anchor pr.**	**#1**				**#2**			**#3**			**#4**		**#6 control**
PCR2†	TCF3-M1r-n+	M1f + M6f + M12f + M13f + M26f + M29f + M33f + M36f				M4f + M8f + M9f + M10f + M16f + M21f + M23f			M2f + M11f + M20f + M25f + M28f + M34f + M35f			M3f + M5f + M14f + M18f + M19f + M24f + M32f		PBX1-M0f + M1r (no anchor)
		#5												
		M7f + M15f + M17f + M22f + M27f + M30f + M31f												

*for MLL fusion proteins multiplexing was done only in the first round of semi-nested PCR.

† for ETV6-RUNX1 and TCF3-PBX1 multiplexing was done for both rounds of PCR. For samples scoring positive, a third validation round using single primers was added.

To avoid contamination by airborne DNA, all PCR reactions were assembled under clean-room conditions in an UV-sterilized PCR cabinet with separate equipment and rooms for pre- and post-PCR procedures.

### Evaluation of results

A sample was scored as PCR-positive if a primer pair specific for a translocation circle yielded a threshold cycle (C_T_) that was clearly decreased compared to the cohort of all other primer pairs. Positive real time products were run on standard agarose gels for determination of size. In addition DNA was isolated from the gel and sequenced from both sides using the PCR amplification primers.

The higher number of primers necessary to cover the t(12;21) and t(1;19) breakpoint region mandated multiplexing also during the second round of PCR. Therefore positively scoring products obtained with a primer pool were re-tested in a third round PCR using single forward primers.

## Results

### Validation of the GIPFEL procedure

**Figure 2:**
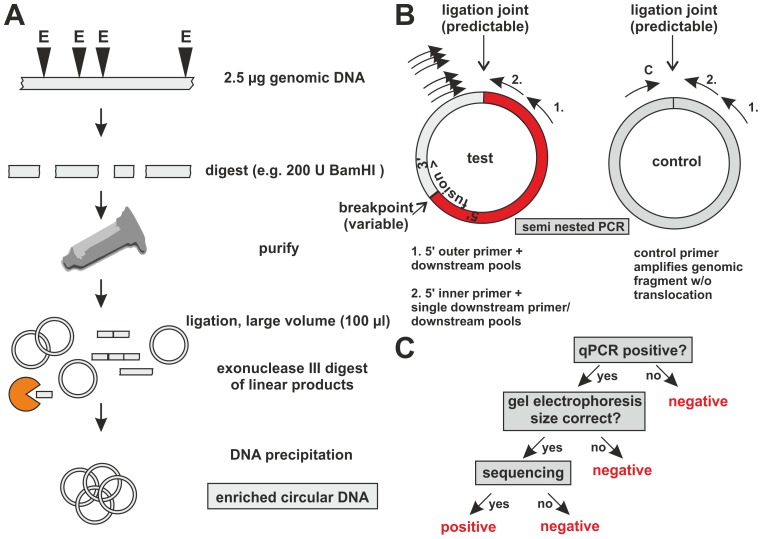
Flow chart of the GIPFEL procedure. A. Biochemical steps for enrichment of circularized DNA. The products of a restriction enzyme (E) digest of genomic material are column purified and ligated in a large volume. Subsequently exonuclease III (presented in yellow) removes remaining linear fragments allowing enrichment for circularized DNA. B. PCR strategy to detect the presence of translocation specific circles. Primer pairs are designed that cover all possible ligation joints of translocation specific ligation products. Semi-nested PCR is performed first with an outer primer corresponding to the 5′ portion of the fusion and pools of downstream primers. The PCR products from these reactions are used as templates for secondary PCRs using a 5′ inner primer and the same downstream primers, yet in different combinations. A control PCR amplifies a ligation joint created from wild-type cells. C. Decision tree for scoring of GIPFEL results.

To generate a genomic DNA preparation enriched in circular ligated DNA a 4-step biochemical procedure was developed (figure 2A). After digestion of genomic DNA and purification of a genome wide population of restriction fragments the nucleic acid was converted to circular form by ligation in a large volume. Remaining linear fragments were removed by digesting with exonuclease III followed by alcohol precipitation to prepare a template for PCR analysis.

PCR was designed in a semi-nested setup (figure 2B) pre-amplifying with an outer anchor primer (three primers for ETV6) binding to sequences of the 5′ fusion portion. This primer was paired with pools of downstream primers corresponding to the predicted 3′ fusion sequence. The reaction products of this primary PCR served as input for the next round of PCR. Secondary PCRs were monitored with SYBR green on a real time machine using a 5′ inner primer (three primers for ETV6) and either each downstream primer in individual combination (for MLL fusion proteins) or again pools of downstream primers (see Table 1 for primer sequences and Table 2 for multiplexing strategies). Primers amplifying a nearby genomic region unaffected by the translocation were employed alongside as controls. For further evaluation amplified PCR products were sized on agarose gels, isolated and sequenced (figure 2C). A sample was scored positive if the size and the predicted sequence of a PCR product could be unequivocally confirmed (see Table S1 for a list of predicted ligation joint sequences).

**Figure 3:**
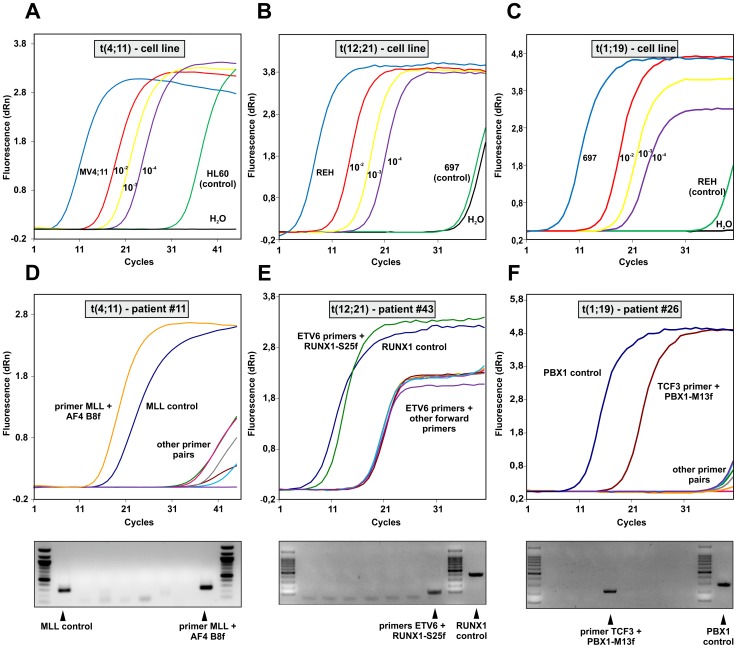
Examples of GIPFEL results. A. Sensitivity test. Circularized genomic DNA was produced from MV4;11 cells a cell line with a known t(4;11) translocation and from HL60 cells as “non-translocation” control as well as from various mixtures “diluting” MV4;11 cells in a population of HL60 as indicated. GIPFEL was performed and real-time amplification curves are shown. B. As in “A” with REH t(12;21) cells and 697 cells instead of HL60 cells. C. As in “A” with 697 t(1;19) and REH cells. D. Example for a GIPFEL result using patient DNA. Upper panel: Amplification chart of a typical GIPFEL experiment with patient DNA. Amplification is achieved with the genomic MLL control primer and a translocation specific primer pair. Lower panel: Agarose gel electrophoresis of the 8 individual secondary PCRs interrogating the (4;11) breakpoint region. E. Results presented as in “D” for a t(12;21) breakpoint. F. Results for a t(1;19) patient sample.

To evaluate the efficiency of the overall process we validated the procedure with DNA from three cell lines: MV4;11 carries a t(4;11), REH contains t(12;21) and 697 was used to detect t(1;19). For all lines the exact location of the breakpoint is known obviating the need for multiplexing in the set-up experiments. DNA from cell lines negative for the translocations to be tested (HL60, 697, REH) served as background control. Translocation bearing cells were mixed in various ratios with control cells and the GIPFEL procedure was performed (figure 3). Under these optimal conditions detection of signature circles was possible for all translocations down to a dilution of 1 into 10^−4^. This dilution is equivalent to a calculated presence of 19 target molecules per PCR reaction (2.5 µg DNA = 3.8×10^5^ cells ×10^−4^ = 38 but because only 50% of the circularization reaction was used as template for PCR, effectively a calculated maximum of 19 template molecules have been present).

**Figure 4:**
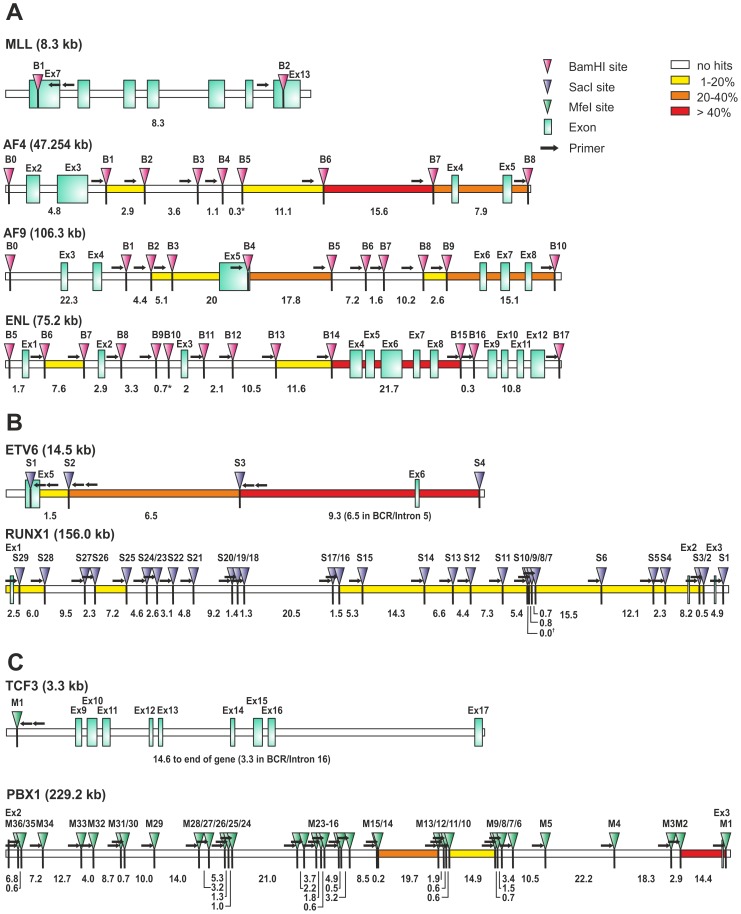
Breakpoint distribution, restriction site and primer locations for individual translocations. A. Schematic depiction of the 11q23 breakpoint region covered by GIPFEL. Consecutively numbered BamHI sites (B), primer locations (arrows) and exons (squares) involved are depicted. Numbers denote the size in kb between restriction sites. * Note: For restriction fragments <1 kb no primers were designed. B. Schematic depiction of the t(12;21) breakpoint regions covered by GIPFEL. SacI sites (S), primer locations and exons involved are depicted as described in A. Numbers denote the size in kb between restriction sites. † Note: Restriction sites S9 and S10 were 4 bp apart. No primer was designed for site S9. C. Schematic depiction of the t(1;19) breakpoint covered by GIPFEL. Presentation as in A and B. Digest was carried out with MfeI (M). The heatmap indicates the frequency of the breakpoints detected in the respective region.

To further validate the method on actual patient samples, DNA was obtained from clinical repositories. A collection was assembled encompassing 21 MLL-AF4, 16 MLL-AF9, 18 MLL-ENL, 60 ETV6-RUNX1, and 30 TCF3-PBX1 cases. Five negative control samples were added to each translocation group and the samples were blinded for processing. Because of the limited amount of the clinical material the procedure was performed with 1 µg of genomic DNA as input for MLL bearing translocations. For the other translocations the DNA was genome amplified and 2.5 µg were used. Again the three-tiered decision process of real-time PCR, agarose gelelectrophoresis and sequencing was applied to score the results. Representative examples of positive experiments are shown in figure 3D–F. Upon unblinding GIPFEL showed 100% specificity as no false positive results were obtained. As expected, accuracy was lower. For MLL-AF4, MLL-AF9, MLL-ENL, ETV6-RUNX1, and TCF3-PBX1, 83%, 65%, 24%, 64% and 39% of positive samples were correctly called. Sensitivity was comparable to cell line experiments. When tested with selected patient material positive samples still could be successfully called at dilutions between 10^−3^ and 10^−4^. A summary of patient and cell line data is given in table 3. Because GIPFEL also gives topical information of the breakpoint location depending on the primer pair yielding a positive readout, a breakpoint distribution chart could be assembled (figure 4). As observed previously, chromosomal junction sites were not randomly distributed but clustered in certain areas corresponding to known hotspots of instability giving additional support to the validity of our GIPFEL results [5], [10]–[20].

**Table 3:** GIPFEL results summary.

MLLAF4 (n = 23) Breakpoint region:	# patient samples + cell lines
**B0–B1**	0
**B1–B2**	2
**B2–B4**	0
**B5–B6**	1
**B6–B7**	9*+1
**B7–B8**	7*+1
**not detected**	3
**MLLAF9 (n = 17)** Breakpoint region:	
**B0–B2**	0
**B2–B3**	1
**B3–B4**	1
**B4–B5**	4*
**B5–B8**	0
**B8–B9**	1
**B9–B10**	5*+1
**not detected**	5
**MLLENL (n = 17)** Breakpoint region:	
**B5–B6**	0
**B6–B7**	1
**B7–B13**	0
**B13–B14**	1
**B14–B15**	2
**B15–B17**	0
**not detected**	13
**ETV6RUNX1 (n = 61)**	
Breakpoint region RUNX1:	
**S1–S2**	0
**S2–S3**	2
**S3–S4**	1
**S4–S5**	3
**S5–S6**	3
**S6–S7**	2
**S7–S8**	0
**S8–S9**	2
**S10–S11**	4
**S11–S12**	3
**S12–S13**	1
**S13–S14**	2+1
**S14–S15**	5
**S15–S16**	1
**S16–S25**	0
**S25–S26**	2
**S26–S28**	0
**S28–S29**	6
**S29–S30**	1
**not detected**	22
Breakpoint Region ETV6:	
**S1–S2**	6*
**S2–S3**	11*+1
**S3–S4**	22
**not detected**	22
**TCF3PBX1 (n = 31)**	
Breakpoint region:	
**M1–M2**	5+1
**M2–M9**	0
**M9–M10**	2
**M10–M13**	0
**M13–M14**	4
**M14–M37**	0
**not detected**	19

* =  two different breakpoints were detected in a patient sample.

## Discussion

Here we present a proof-of-principle study demonstrating that it is possible to detect the most commonly occurring translocations in childhood leukemia using small amounts of DNA without having to resort to long range PCR or unstable RNA. The GIPFEL method relies on the prior knowledge of the genomic region where breaks occur. As long as this information is available it can be adapted to any recurrent translocation. At the same time this is also a drawback of the technique. Breaks outside of the pre-defined genomic region will not be detected. Likewise, more complicated genomic rearrangements might elude discovery because they alter the predicted ligation joints. Translocations resulting from more complicated reshuffling of the genome have been described [21]. During our study we serendipitously detected at (11;19) breakpoint where material of chromosome 5 had been interspersed at the junction site of chromosome 11 and 19 (not shown). Events of this type are the most likely explanation for the false negative rate in the present study. In addition the fact that occasionally only one of two closely spaced restriction sites was covered by primer pairs also causes small “blind spots”. However, compared to the size of most breakpoint regions it is highly improbable that these tiny regions <1 kb should have a major impact on the sensitivity of the assay.

The biochemical preparation of circular ligated DNA seems to be close to the optimum. Reactions that contained less than 20 calculated template molecules still yielded a positive readout indicating that all previous preparatory steps worked with near perfect efficiency. Therefore the sensitivity of GIPFEL seems to be mainly limited by the amount of total template DNA that can be fed per PCR reaction. This restricts the practical threshold of GIPFEL to about 1 in 10^4^ cells which falls in the range of most DNA based methods. We estimate this sensitivity should suffice to discover most clinically meaningful cases.

Another current constraint is the number of PCR reactions that need to be manually assembled to cover a translocation region. However, for this aspect improvements are in sight as new developments like digital droplet PCR should be easily adaptable to GIPFEL allowing the simultaneous screening for multiple translocations in a high-throughput fashion. Despite the fact that t(11;19) and t(1;19) do not read out optimally in our assay, most cases of the much more frequently occurring t(4;11), t(9;11) and particularly t(12;21) will be recorded. In addition actual population based frequencies of the less easily detectable translocations may be extrapolated from the incidence as detected by GIPFEL corrected by the respective accuracy rate. In addition it is to be expected that NGS data from actual breakpoint regions will beome increasingly available. This information will aid in developing better primers for GIPFEL thus increasing precision of this method.

In summary GIPFEL could become a valuable tool particularly in prospective settings. Patients that have been exposed to topoisomerase inhibitors during the treatment of non-blood related neoplastic diseases are at a higher risk developing 11q23 translocation-positive secondary malignancies. Similarly, persons exposed to ionizing radiation might be screened for the appearance of translocation positive clones. Finally, GIPFEL may be used to solve the ongoing scientific discussion about the actual frequency of pre-leukemic events in healthy newborns, who never develop leukemia in later life. For this purpose birth cohorts might be screened for the presence of interchromosomal fusion sequences in apparently healthy newborns. Previous studies gave highly divergent results ranging from 1∶100 ETV6-RUNX1 positive cases [22] to less than 1 in 1417 cord blood samples [23], [24]. In all these cases GIPFEL may detect the appearance of translocation positive clones allowing for follow up and maybe early treatment.

## Supporting Information

Table S1 **Predicted joining sequences for each primer combination.**
(DOCX)
