# Supplementary material for: Genomic Inverse PCR for Exploration of Ligated Breakpoints (GIPFEL), a New Method to Detect Translocations in Leukemia
Source: PLoS One. 2014 Aug 19;9(8):e104419. doi: 10.1371/journal.pone.0104419 (PMC4138100; doi:10.1371/journal.pone.0104419)
Supplement: Table S1 — Predicted joining sequences for each primer combination. (DOCX) [file pone.0104419.s001.docx]

Supplemental table S1: Predicted joining sequences for each primer combination

| primer pair | AF4_restriction site_MLL |
| --- | --- |
| MLL-B1r-n + |  |
| AF4-B1f | AAAAAATAGATACACCTATA **GGATCC** TGCCCCAAAGAAAAGCAG |
| AF4-B2f | AGAACTCCGCGATCTCACGT **GGATCC** TGCCCCAAAGAAAAGCAG |
| AF4-B3f | TTGGGAGAAGCACAGGAGTG **GGATCC** TGCCCCAAAGAAAAGCAG |
| AF4-B4f.2 | GATGTTTGTGGATGGTTATT **GGATCC** TGCCCCAAAGAAAAGCAG |
| AF4-B6f | ATCCTCATCCCCTAGAGCCA **GGATCC** TGCCCCAAAGAAAAGCAG |
| AF4-B7f | ATGCTAGAGATGATTCCAGT **GGATCC** TGCCCCAAAGAAAAGCAG |
| AF4-B8f | CAATACGTTCTCTCATTTGA **GGATCC** TGCCCCAAAGAAAAGCAG |
|  | **AF9_restriction site_MLL** |
| MLL-B1r-n + |  |
| AF9-B1f.2 | ATATGACCAGCAAGGTAGTT **GGATCC** TGCCCCAAAGAAAAGCAG |
| AF9-B2f.3 | GGACAGATTGAGTGAGATAA **GGATCC** TGCCCCAAAGAAAAGCAG |
| AF9-B3f | CCTTTATTTTGATAGCAAAA **GGATCC** TGCCCCAAAGAAAAGCAG |
| AF9-B4f | CCGCCATTTGATGATATTGT **GGATCC** TGCCCCAAAGAAAAGCAG |
| AF9-B5f | CCTATGCTGTCCTGAACACA **GGATCC** TGCCCCAAAGAAAAGCAG |
| AF9-B6f.2 | TCGTGAGAAACTGTCTTGAA **GGATCC** TGCCCCAAAGAAAAGCAG |
| AF9-B7f | CTGCCAACTCTTGCTATAGA **GGATCC** TGCCCCAAAGAAAAGCAG |
| AF9-B8f | ATTCTCAAAGCTCTTAACAA **GGATCC** TGCCCCAAAGAAAAGCAG |
| AF9-B9f | CTCAAACTCCTGGGCTCAGG **GGATCC** TGCCCCAAAGAAAAGCAG |
| AF9-B10f | CTGGTTAACTAGAAAATTTG **GGATCC** TGCCCCAAAGAAAAGCAG |
|  | **ENL_restriction site_MLL** |
| MLL-B1r-n + |  |
| ENL-B6f | TTGGGCCCTCTCCGCCCCTG **GGATCC** TGCCCCAAAGAAAAGCAG |
| ENL-B7f | TGGGAAGTCTCAGGAGCAGA **GGATCC** TGCCCCAAAGAAAAGCAG |
| ENL-B8f.2 | TGTGAGCCTGTTTACCTTTG **GGATCC** TGCCCCAAAGAAAAGCAG |
| ENL-B9f | CCTCTCTTTTTTTCTTTCTG **GGATCC** TGCCCCAAAGAAAAGCAG |
| ENL-B11f | TGCACTTCAAAAACAAGGTA **GGATCC** TGCCCCAAAGAAAAGCAG |
| ENL-B12f | AATCCCTGCCTGCTCTTTTG **GGATCC** TGCCCCAAAGAAAAGCAG |
| ENL-B13f.2 | GGCTCCAGCTCTTCAAGGGG **GGATCC** TGCCCCAAAGAAAAGCAG |
| ENL-B14f | CTCGAACTCCTGGGCTTAAG **GGATCC** TGCCCCAAAGAAAAGCAG |
| ENL-B15f.7 | CCCTGGGAGGGCCAAAGCAG **GGATCC** TGCCCCAAAGAAAAGCAG |
| ENL-B16f.2 | ACCTGCCTTACATGGGGCGA **GGATCC** TGCCCCAAAGAAAAGCAG |
| ENL-B17f | GTCCTGAGGACTCTTCCTTG **GGATCC** TGCCCCAAAGAAAAGCAG |
|  | **RUNX1_restriction site_ETV6** |
| ETV6-S1r-n + |  |
| RUNX1-S1f | CTACTAAAAATCCAAATACA **GAGCTC** AGGGACCCAGGCCGCACC |
| RUNX1-S2f | GACCGGGGGCGGGGCCCTCC **GAGCTC** AGGGACCCAGGCCGCACC |
| RUNX1-S3f | CGCCGTCGGGGGCGTTCCGG **GAGCTC** AGGGACCCAGGCCGCACC |
| RUNX1-S4f | GTATGTGGCTTCCTGCTACT **GAGCTC** AGGGACCCAGGCCGCACC |
| RUNX1-S5f | GCTTTTGTAATATTGTTTCC **GAGCTC** AGGGACCCAGGCCGCACC |
| RUNX1-S6f | TTGCATCGGTTTGGAATGTT **GAGCTC** AGGGACCCAGGCCGCACC |
| RUNX1-S7f | AAGAACTCAGTATAAACCTA **GAGCTC** AGGGACCCAGGCCGCACC |
| RUNX1-S8f | AGAATTTAAGAGAGAAGGGG **GAGCTC** AGGGACCCAGGCCGCACC |
| RUNX1-S10f | TACCTGGATGGCAGCTTCAG **GAGCTC** AGGGACCCAGGCCGCACC |
| RUNX1-S11f | AAATCATGGCAGTGGAAGGA **GAGCTC** AGGGACCCAGGCCGCACC |
| RUNX1-S12f | AATAATGGACACTTGTACCT **GAGCTC** AGGGACCCAGGCCGCACC |
| RUNX1-S13f | ACGAGCTTGCCTTTTCTCTG **GAGCTC** AGGGACCCAGGCCGCACC |
| RUNX1-S14f | GTGGCCCAGGATGGCTGCCT **GAGCTC** AGGGACCCAGGCCGCACC |
| RUNX1-S15f | ATTTGAGACCCTGCTAGGGA **GAGCTC** AGGGACCCAGGCCGCACC |
| RUNX1-S16f | TTCCATGAAGTACACAACAA **GAGCTC** AGGGACCCAGGCCGCACC |
| RUNX1-S17f | AAGGTCCAGCCAAACACAGT **GAGCTC** AGGGACCCAGGCCGCACC |
| RUNX1-S18f | CCTGTTCACGGGCCTGTCTG **GAGCTC** AGGGACCCAGGCCGCACC |
| RUNX1-S19f | TAGGCAGAGTTATCACACCT **GAGCTC** AGGGACCCAGGCCGCACC |
| RUNX1-S20f | AAAGCATCTCCACACAGAAA **GAGCTC** AGGGACCCAGGCCGCACC |
| RUNX1-S21f | GACTGTCCTGGGTGAATCAG **GAGCTC** AGGGACCCAGGCCGCACC |
| RUNX1-S22f | ATTCCACTTGACAGTCAGCA **GAGCTC** AGGGACCCAGGCCGCACC |
| RUNX1-S23f | CCGTCTGTGGCTGGAAAGCA **GAGCTC** AGGGACCCAGGCCGCACC |
| RUNX1-S24f | CGGCACCCTTTGTTCACTCG **GAGCTC** AGGGACCCAGGCCGCACC |
| RUNX1-S25f | TTTTGGAAGCATTGAGAGAA **GAGCTC** AGGGACCCAGGCCGCACC |
| RUNX1-S26f | TGGTGGAGAGAGGCCACCAA **GAGCTC** AGGGACCCAGGCCGCACC |
| RUNX1-S27f | TTCCCACCAAGAGTGTGCAA **GAGCTC** AGGGACCCAGGCCGCACC |
| RUNX1-S28f | AAAAGAGCAAACAGAAGTCA **GAGCTC** AGGGACCCAGGCCGCACC |
| RUNX1-S29f | AGGCAGTGTAGGGAGGGCCT **GAGCTC** AGGGACCCAGGCCGCACC |
|  | **RUNX1_restriction site_ETV6** |
| ETV6-S2r-n + |  |
| RUNX1-S1f | CTACTAAAAATCCAAATACA **GAGCTC** TAGCTGACCATCAAGGTG |
| RUNX1-S2f | GACCGGGGGCGGGGCCCTCC **GAGCTC** TAGCTGACCATCAAGGTG |
| RUNX1-S3f | CGCCGTCGGGGGCGTTCCGG **GAGCTC** TAGCTGACCATCAAGGTG |
| RUNX1-S4f | GTATGTGGCTTCCTGCTACT **GAGCTC** TAGCTGACCATCAAGGTG |
| RUNX1-S5f | GCTTTTGTAATATTGTTTCC **GAGCTC** TAGCTGACCATCAAGGTG |
| RUNX1-S6f | TTGCATCGGTTTGGAATGTT **GAGCTC** TAGCTGACCATCAAGGTG |
| RUNX1-S7f | AAGAACTCAGTATAAACCTA **GAGCTC** TAGCTGACCATCAAGGTG |
| RUNX1-S8f | AGAATTTAAGAGAGAAGGGG **GAGCTC** TAGCTGACCATCAAGGTG |
| RUNX1-S10f | TACCTGGATGGCAGCTTCAG **GAGCTC** TAGCTGACCATCAAGGTG |
| RUNX1-S11f | AAATCATGGCAGTGGAAGGA **GAGCTC** TAGCTGACCATCAAGGTG |
| RUNX1-S12f | AATAATGGACACTTGTACCT **GAGCTC** TAGCTGACCATCAAGGTG |
| RUNX1-S13f | ACGAGCTTGCCTTTTCTCTG **GAGCTC** TAGCTGACCATCAAGGTG |
| RUNX1-S14f | GTGGCCCAGGATGGCTGCCT **GAGCTC** TAGCTGACCATCAAGGTG |
| RUNX1-S15f | ATTTGAGACCCTGCTAGGGA **GAGCTC** TAGCTGACCATCAAGGTG |
| RUNX1-S16f | TTCCATGAAGTACACAACAA **GAGCTC** TAGCTGACCATCAAGGTG |
| RUNX1-S17f | AAGGTCCAGCCAAACACAGT **GAGCTC** TAGCTGACCATCAAGGTG |
| RUNX1-S18f | CCTGTTCACGGGCCTGTCTG **GAGCTC** TAGCTGACCATCAAGGTG |
| RUNX1-S19f | TAGGCAGAGTTATCACACCT **GAGCTC** TAGCTGACCATCAAGGTG |
| RUNX1-S20f | AAAGCATCTCCACACAGAAA **GAGCTC** TAGCTGACCATCAAGGTG |
| RUNX1-S21f | GACTGTCCTGGGTGAATCAG **GAGCTC** TAGCTGACCATCAAGGTG |
| RUNX1-S22f | ATTCCACTTGACAGTCAGCA **GAGCTC** TAGCTGACCATCAAGGTG |
| RUNX1-S23f | CCGTCTGTGGCTGGAAAGCA **GAGCTC** TAGCTGACCATCAAGGTG |
| RUNX1-S24f | CGGCACCCTTTGTTCACTCG **GAGCTC** TAGCTGACCATCAAGGTG |
| RUNX1-S25f | TTTTGGAAGCATTGAGAGAA **GAGCTC** TAGCTGACCATCAAGGTG |
| RUNX1-S26f | TGGTGGAGAGAGGCCACCAA **GAGCTC** TAGCTGACCATCAAGGTG |
| RUNX1-S27f | TTCCCACCAAGAGTGTGCAA **GAGCTC** TAGCTGACCATCAAGGTG |
| RUNX1-S28f | AAAAGAGCAAACAGAAGTCA **GAGCTC** TAGCTGACCATCAAGGTG |
| RUNX1-S29f | AGGCAGTGTAGGGAGGGCCT **GAGCTC** TAGCTGACCATCAAGGTG |
|  | **RUNX1_restriction site_ETV6** |
| ETV6-S3r-n + |  |
| RUNX1-S1f | CTACTAAAAATCCAAATACA **GAGCTC** CTGGAGACAGATGGCCCC |
| RUNX1-S2f | GACCGGGGGCGGGGCCCTCC **GAGCTC** CTGGAGACAGATGGCCCC |
| RUNX1-S3f | CGCCGTCGGGGGCGTTCCGG **GAGCTC** CTGGAGACAGATGGCCCC |
| RUNX1-S4f | GTATGTGGCTTCCTGCTACT **GAGCTC** CTGGAGACAGATGGCCCC |
| RUNX1-S5f | GCTTTTGTAATATTGTTTCC **GAGCTC** CTGGAGACAGATGGCCCC |
| RUNX1-S6f | TTGCATCGGTTTGGAATGTT **GAGCTC** CTGGAGACAGATGGCCCC |
| RUNX1-S7f | AAGAACTCAGTATAAACCTA **GAGCTC** CTGGAGACAGATGGCCCC |
| RUNX1-S8f | AGAATTTAAGAGAGAAGGGG **GAGCTC** CTGGAGACAGATGGCCCC |
| RUNX1-S10f | TACCTGGATGGCAGCTTCAG **GAGCTC** CTGGAGACAGATGGCCCC |
| RUNX1-S11f | AAATCATGGCAGTGGAAGGA **GAGCTC** CTGGAGACAGATGGCCCC |
| RUNX1-S12f | AATAATGGACACTTGTACCT **GAGCTC** CTGGAGACAGATGGCCCC |
| RUNX1-S13f | ACGAGCTTGCCTTTTCTCTG **GAGCTC** CTGGAGACAGATGGCCCC |
| RUNX1-S14f | GTGGCCCAGGATGGCTGCCT **GAGCTC** CTGGAGACAGATGGCCCC |
| RUNX1-S15f | ATTTGAGACCCTGCTAGGGA **GAGCTC** CTGGAGACAGATGGCCCC |
| RUNX1-S16f | TTCCATGAAGTACACAACAA **GAGCTC** CTGGAGACAGATGGCCCC |
| RUNX1-S17f | AAGGTCCAGCCAAACACAGT **GAGCTC** CTGGAGACAGATGGCCCC |
| RUNX1-S18f | CCTGTTCACGGGCCTGTCTG **GAGCTC** CTGGAGACAGATGGCCCC |
| RUNX1-S19f | TAGGCAGAGTTATCACACCT **GAGCTC** CTGGAGACAGATGGCCCC |
| RUNX1-S20f | AAAGCATCTCCACACAGAAA **GAGCTC** CTGGAGACAGATGGCCCC |
| RUNX1-S21f | GACTGTCCTGGGTGAATCAG **GAGCTC** CTGGAGACAGATGGCCCC |
| RUNX1-S22f | ATTCCACTTGACAGTCAGCA **GAGCTC** CTGGAGACAGATGGCCCC |
| RUNX1-S23f | CCGTCTGTGGCTGGAAAGCA **GAGCTC** CTGGAGACAGATGGCCCC |
| RUNX1-S24f | CGGCACCCTTTGTTCACTCG **GAGCTC** CTGGAGACAGATGGCCCC |
| RUNX1-S25f | TTTTGGAAGCATTGAGAGAA **GAGCTC** CTGGAGACAGATGGCCCC |
| RUNX1-S26f | TGGTGGAGAGAGGCCACCAA **GAGCTC** CTGGAGACAGATGGCCCC |
| RUNX1-S27f | TTCCCACCAAGAGTGTGCAA **GAGCTC** CTGGAGACAGATGGCCCC |
| RUNX1-S28f | AAAAGAGCAAACAGAAGTCA **GAGCTC** CTGGAGACAGATGGCCCC |
| RUNX1-S29f | AGGCAGTGTAGGGAGGGCCT **GAGCTC** CTGGAGACAGATGGCCCC |
|  | **PBX1_restriction site_TCF3** |
| TCF3-M1r-n + |  |
| PBX1-M1f | CACTTTACCCAGACTCAGTT **CAATTG** GGGCTCCCTGTTACATCC |
| PBX1-M2f | GCTTGCTATCAACCTTGCTA **CAATTG** GGGCTCCCTGTTACATCC |
| PBX1-M3f | GCTTAATTGTGATGATATCA **CAATTG** GGGCTCCCTGTTACATCC |
| PBX1-M4f | CAGTGTTTTCCATCTGCAGT **CAATTG** GGGCTCCCTGTTACATCC |
| PBX1-M5f | GAACAAAGCTGAAAAGAGAG **CAATTG** GGGCTCCCTGTTACATCC |
| PBX1-M6f | TTTACAATGTACTCTGCAGA **CAATTG** GGGCTCCCTGTTACATCC |
| PBX1-M7f | TAATGACTTAAAAGCGAAAA **CAATTG** GGGCTCCCTGTTACATCC |
| PBX1-M8f | TACTTCCCTACATTTTATGA **CAATTG** GGGCTCCCTGTTACATCC |
| PBX1-M9f | GAGAGAGAACAAATGGTTAG **CAATTG** GGGCTCCCTGTTACATCC |
| PBX1-M10f | TTTATTGAACAAGACTTTTA **CAATTG** GGGCTCCCTGTTACATCC |
| PBX1-M11f | AAGTCCCTTCTCCACACATC **CAATTG** GGGCTCCCTGTTACATCC |
| PBX1-M12f | TCAAACTGAGTGTGGACACC **CAATTG** GGGCTCCCTGTTACATCC |
| PBX1-M13f | TGCCATTTTTTGAACTTCAC **CAATTG** GGGCTCCCTGTTACATCC |
| PBX1-M14f | ATAGAAAGTAAATGAAAACT **CAATTG** GGGCTCCCTGTTACATCC |
| PBX1-M15f | ACTAAGCATTTAAAATAAAT **CAATTG** GGGCTCCCTGTTACATCC |
| PBX1-M16f | CTCCTGCTATCTTAAAGCCA **CAATTG** GGGCTCCCTGTTACATCC |
| PBX1-M17f | TTTAGAATGTACACACTGCC **CAATTG** GGGCTCCCTGTTACATCC |
| PBX1-M18f | AGGCTATGATTCTATATGAG **CAATTG** GGGCTCCCTGTTACATCC |
| PBX1-M19f | CCGTACCAAAACTGTCACCA **CAATTG** GGGCTCCCTGTTACATCC |
| PBX1-M20f | TGGCGCCTTAAATAAAATAT **CAATTG** GGGCTCCCTGTTACATCC |
| PBX1-M21f | TACACTTTCATGGAGGGCAT **CAATTG** GGGCTCCCTGTTACATCC |
| PBX1-M22f | TCTGCAGCCACTGTTTAGCT **CAATTG** GGGCTCCCTGTTACATCC |
| PBX1-M23f | TTATATAGACAGTTAGCCTT **CAATTG** GGGCTCCCTGTTACATCC |
| PBX1-M24f | TTGCCTGCCTGTAGAGATGG **CAATTG** GGGCTCCCTGTTACATCC |
| PBX1-M25f | ATGTAATTTAATCTTCCATT **CAATTG** GGGCTCCCTGTTACATCC |
| PBX1-M26f | ATGTGTTTGGGGTTTTTTCT **CAATTG** GGGCTCCCTGTTACATCC |
| PBX1-M27f | TGGTACCGATGTGTGGCCGG **CAATTG** GGGCTCCCTGTTACATCC |
| PBX1-M28f | ATTTACTGATGTTTTTTAAG **CAATTG** GGGCTCCCTGTTACATCC |
| PBX1-M29f | TGTCAGGTTTAGCATTGAGG **CAATTG** GGGCTCCCTGTTACATCC |
| PBX1-M30f | ATTTAGTCTGTTTGGTCTTA **CAATTG** GGGCTCCCTGTTACATCC |
| PBX1-M31f | TCTTTCTTCGGGCAAATCTG **CAATTG** GGGCTCCCTGTTACATCC |
| PBX1-M32f | GGAAGCTTGGATGGTTAGAG **CAATTG** GGGCTCCCTGTTACATCC |
| PBX1-M33f | TTTGTTAACCTATACTGTCA **CAATTG** GGGCTCCCTGTTACATCC |
| PBX1-M34f | TTGAGCTGGTAGTTCTGTAG **CAATTG** GGGCTCCCTGTTACATCC |
| PBX1-M35f | TAGATAGCAATGAATAGCTG **CAATTG** GGGCTCCCTGTTACATCC |
| PBX1-M36f | AGAAGGTGCCATCTGAATTA **CAATTG** GGGCTCCCTGTTACATCC |
